# 新辅助免疫治疗联合化疗在可切除NSCLC中的应用

**DOI:** 10.3779/j.issn.1009-3419.2023.102.40

**Published:** 2023-11-20

**Authors:** Huaiyong WANG, Yun HAN

**Affiliations:** 110000 沈阳，中国医科大学附属盛京医院胸外科; Department of Thoracic Surgery, Shengjing Hospital of China Medical University, Shenyang 110000, China

**Keywords:** 肺肿瘤, 新辅助治疗, 免疫治疗, 治疗周期, 无事件生存期, 手术, Lung neoplasms, Neoadjuvant therapy, Immunotherapy, Treatment cycle, Event-free survival, Surgery

## Abstract

**背景与目的:**

对于可切除的非小细胞肺癌（non-small cell lung cancer, NSCLC），CheckMate-816研究表明，相比于新辅助化疗，免疫治疗联合化疗可将完全病理缓解率（complete pathologic response, pCR）提高21.8%，且无事件生存期（event-free survival, EFS）可显著获益。本研究旨在探讨真实世界中此方法的安全性及可行性。

**方法:**

回顾性分析两中心新辅助免疫治疗联合化疗或单独化疗后接受手术的NSCLC患者的临床数据，并根据治疗周期对免疫治疗联合化疗组进行亚组分析。主要研究终点为EFS及主要病理缓解（major pathologic response, MPR），次要研究终点为pCR、总生存（overall survival, OS）率、治疗相关不良事件（treatment-related adverse events, TRAEs）及手术相关指标。

**结果:**

截至2023年4月，共纳入89例患者，其中联合治疗组54例，单独化疗组35例。联合治疗组及单独化疗组分别有31例（57.4%）及5例（14.3%）患者达到MPR（OR=8.09, 95%CI: 2.72-24.04, P<0.001），联合治疗组中有25例（46.3%）患者达到pCR，单独化疗组中无pCR患者（P<0.001）。中位随访时间为22.1个月。24个月时，两组EFS率分别为77.0%及56.7%（P=0.026），OS率分别为87.1%及67.7%（P=0.020）。联合治疗组中，1-2个周期与3-5个周期组在手术时间、术中失血量、术后并发症等方面无统计学差异。

**结论:**

术前应用免疫治疗联合化疗较单独化疗更有效且未增加手术风险。新辅助免疫治疗联合化疗用药周期的增加对手术难度无明显影响。

## Abstract (en)

**Background and objective:**

For resectable non-small cell lung cancer (NSCLC), the CheckMate-816 study demonstrated that neoadjuvant chemoimmunotherapy increased the rate of complete pathologic response (pCR) by 21.8% compared with chemotherapy alone and resulted in a significant benefit in event-free survival (EFS). This study aimed to investigate the safety and feasibility of this approach in the real world.

**Methods:**

Clinical data from patients with NSCLC who underwent surgery after neoadjuvant chemoimmunotherapy or chemotherapy alone in two centers were analyzed retrospectively, and subgroup analyses were performed for the chemoimmunotherapy group according to treatment cycle. The primary study endpoints were EFS and major pathologic response (MPR), and the secondary study endpoints were pCR, overall survival (OS), treatment-related adverse events (TRAEs), and surgery-related metrics.

**Results:**

As of April 2023, 89 patients had been enrolled, including 54 in the chemoimmunotherapy group and 35 in the chemotherapy alone group. MPR was achieved in 31 (57.4%) and 5 (14.3%) patients in the chemoimmunotherapy group and chemotherapy alone group, respectively (OR=8.09, 95%CI: 2.72-24.04, P<0.001); pCR was achieved in 25 (46.3%) patients in the chemoimmunotherapy group and no patient in the chemotherapy alone group (P<0.001). The median follow-up time was 22.1 months. At 24 months, the EFS rates of the chemoimmunotherapy group and the chemotherapy alone group were 77.0% and 56.7%, respectively (P=0.026), and the OS rates were 87.1% and 67.7%, respectively (P=0.020). In the neoadjuvant chemoimmunotherapy group, there was no significant difference between the 1-2 cycles and 3-5 cycles groups in terms of operation time, intraoperative blood loss, and postoperative complications.

**Conclusion:**

Neoadjuvant chemoimmunotherapy was more effective than chemotherapy alone and did not increase the risk associated with surgery. An increase in the number of cycles of neoadjuvant chemoimmunotherapy had no significant effect on the difficulty of surgery.

肺癌是世界范围内发病率最高的恶性肿瘤之一，并以18%的死亡率成为恶性肿瘤患者最主要的死亡原因^[1]^。根治性手术仍是治疗IA-IIIA期非小细胞肺癌（non-small cell lung cancer, NSCLC）的主要方式，但由于残留肿瘤组织或细胞及微小转移灶的存在^[2]^，在接受手术的患者中仍有20%-70%的比例复发^[3]^。因此对于局部晚期NSCLC患者而言术前接受新辅助治疗至关重要。研究^[4]^显示术前化疗仅能提高术后5%-6%的5年总生存期（overall survival, OS），而针对肿瘤程序性死亡受体1（programmed cell death protein 1, PD-1）或其配体（programmed cell death ligand 1, PD-L1）的免疫检查点抑制剂（immune checkpoint inhibitors, ICIs）在晚期NSCLC中的优异表现为临床医生提供了新思路。CheckMate-159首次报道在新辅助应用Nivolumab后主要病理缓解（major pathologic response, MPR）率达到45%，18个月时无事件生存（event-free survival, EFS）率达到73%^[5]^。III期随机对照研究（randomized controlled trial, RCT）CheckMate-816显示联合治疗组的中位EFS为31.6个月，相比于单独化疗组延长近1年，其完全病理缓解率（complete pathologic response, pCR）也有显著提升（24.0% vs 2.2%）^[6]^。近些年，其他关于术前应用ICIs联合化疗的报道层出不穷，但MPR率为50%-65.4%^[7⇓⇓⇓-11]^，高于CheckMate-816，基于此，本研究旨在探讨真实世界中新辅助免疫治疗联合化疗的安全性及有效性。

## 1 资料与方法

### 1.1 患者资料

本研究回顾性分析了2019年1月至2023年4月中国医科大学附属盛京医院及鞍山市肿瘤医院接受新辅助免疫治疗联合化疗及单独化疗后行手术的IB-IIIC期NSCLC患者[根据美国癌症联合委员会第8版肺癌肿瘤原发灶-淋巴结-转移（tumor-node-metastasis, TNM）分期]。纳入标准如下：（1）18岁以上且美国东部肿瘤协作组（Eastern Cooperative Oncology Group, ECOG）评分在0-1分之间；（2）接受治疗之前均在计算机断层扫描（computed tomography, CT）引导下行细针穿刺活检术或经气管镜取得病理组织并被诊断为NSCLC；（3）接受正电子发射计算机断层扫描（positron emission tomography/CT, PET/CT）、头CT、腹部CT，必要时通过超声支气管镜穿刺确定可疑肿大淋巴结是否受累从而确定肿瘤分期；（4）每21天接受新辅助治疗；（5）具有正常的器官功能并耐受手术。排除标准：（1）表皮生长因子受体（epidermal growth factor receptor, EGFR）或间变性淋巴瘤激酶（anaplastic lymphoma kinase, ALK）突变阳性；（2）曾接受其他抗肿瘤治疗；（3）免疫缺陷病史、免疫抑制剂治疗史、自身免疫疾病史；（4）患有传染性疾病；（5）其他恶性肿瘤病史。

共有89例患者入组，其中联合治疗组中纳入54例，单独化疗组中纳入35例。两组患者临床基线特征基本平衡良好，中位周期数为2（IQR: 2-3）（表1）。

**表1:** 纳入患者的基线特征

Characteristic	Total (n=89)	ICIs+Chemo (n=54)	Chemo (n=35)	P
Age, n (%)				
Median (range)	61 (44-78)	62 (45-76)	60 (44-78)	0.421
<60 yr	38 (42.7)	21 (38.9)	17 (48.6)	0.367
≥60 yr	51 (57.3)	33 (61.1)	18 (51.4)	
Gender, n (%)				0.103
Male	69 (77.5)	45 (83.3)	24 (68.6)	
Female	20 (22.5)	9 (16.7)	11 (31.4)	
Smoking history, n (%)				0.142
Yes	59 (66.3)	39 (72.2)	20 (57.1)	
No	30 (33.7)	15 (27.8)	15 (42.9)	
Tumor status, n (%)†				0.144
cT1	11 (12.4)	7 (13.0)	4 (11.4)	
cT2	33 (37.1)	16 (29.6)	17 (48.6)	
cT3	28 (31.5)	18 (33.3)	10 (28.6)	
cT4	17 (19.1)	13 (24.1)	4 (11.4)	
Lymph node status, n (%)†				0.210
N0	15 (16.9)	8 (14.8)	7 (20.0)	
N1	21 (23.6)	12 (22.2)	9 (25.7)	
N2	48 (53.9)	29 (53.7)	19 (54.3)	
N3	5 (5.6)	5 (9.3)	0 (0.0)	
Disease stage, n (%)†				0.080
I	6 (6.7)	2 (3.7)	4 (11.4)	
II	16 (18.0)	8 (14.8)	8 (22.9)	
III	67 (75.3)	44 (81.5)	23 (65.7)	
Histology type, n (%)				0.092
Squamous	67 (75.3)	44 (81.5)	23 (65.7)	
Non squamous	22 (24.7)	10 (18.5)	12 (34.3)	
Type of ICIs, n (%)				<0.001
Tislelizumab	24 (27.0)	24 (44.4)	0 (0.0)	
Sintilimab	21 (23.6)	21 (38.9)	0 (0.0)	
Camrelizumab	9 (10.1)	9 (16.7)	0 (0.0)	
Cycles				
Median (IQR)	2 (2, 3)	2 (2, 3)	2 (2, 3)	0.035
PD-L1 expression, n (%)				0.581
≥1%	42 (47.2)	27 (50.0)	15 (42.9)	
<1%	37 (41.6)	20 (37.0)	17 (48.6)	
NA	10 (11.2)	7 (13.0)	3 (8.5)	

†The tumor-node-metastasis (TNM) classification of malignant tumors, 8^th^ edition, was used for classification. Chemo: chemotherapy; ICIs: immune checkpoint inhibitors; NA: not applicable; IQR: interquartile range; PD-L1: programmed cell death ligand 1.

### 1.2 新辅助治疗

回顾性收集了患者的基本信息、新辅助用药方案、周期、病理类型及分期并按照治疗方案的不同分为联合治疗组及单独化疗组。肿瘤分期由2名医生共同确定，研究中涉及的患者用药方案是由胸外科专家及肿瘤内科专家通过多学科会诊共同探讨研究制定。患者每21天入院接受相应治疗方案并在每个周期的治疗前后接受血液细胞学及血液生化学检测。

### 1.3 手术

术前7天内行PET/CT、头CT、腹部CT、全身骨放射性核素断层扫描（emission computed tomography, ECT）判断肿瘤影像学反应及可切除性。手术入路及术式由胸外科专家共同制定，同时通过手术相关记录等回顾性收集相关指标：手术入路、术式、手术时间、估计术中出血量、是否中转开胸及原因、手术切缘、术后并发症及30 d的死亡率、胸腔闭式引流持续时间及术后住院时间。手术时间定义为从切开皮肤到切口缝合完毕的时间，术后并发症根据胸外科协会标准定义。术后辅助应用免疫联合化疗、单独免疫治疗、放疗或化疗等。

### 1.4 研究终点

随访截止于2023年7月，本研究主要通过比较两种治疗方案来探讨其安全性及有效性。主要研究终点为EFS及MPR。EFS定义为从新辅助治疗开始到术后疾病进展（progressive disease, PD）或死亡的时间；MPR定义为切除标本中原发肿瘤灶及淋巴结残留的活肿瘤细胞比例≤10%。次要研究终点为pCR、OS、治疗相关不良事件（treatment-related adverse events, TRAEs）及手术相关指标。pCR定义为切除标本中原发肿瘤灶及淋巴结残留的活肿瘤细胞比例为0；根据实体瘤疗效评估标准（Response Evaluation Criteria in Solid Tumors 1.1, RECIST 1.1）^[12]^评估肿瘤影像学变化；依据常见不良反应术语评定标准（Common Terminology Criteria for Adverse Events, CTCAE）5.0版本对TRAEs进行分级；OS定义为从新辅助治疗开始到术后死亡的时间；定义末次新辅助治疗到手术的间隔时间大于37 d为手术延迟。

### 1.5 统计分析

54例患者接受免疫治疗联合化疗，35例患者接受单独化疗，我们假设单独化疗组及联合治疗组中MPR率分别为10%与47%^[6]^，使用双侧Z检验计算此项研究可提供90%以上的检验效能（α水平为0.01）。

统计分析均使用SPSS 26.0版完成。连续变量表示为均数±标准差或中位数及其范围或四分位数间距，分类变量表示为计数及百分比。对于连续性变量使用Student’s t检验，对分类变量使用卡方检验或Fisher精确检验，对于等级变量或不服从正态分布数据采用Mann-Whitney U检验。采用分层Cochran-Mantel-Haenszel检验比较两组患者的MPR。使用Graphpad prism 9.5绘制Kaplan-Meier曲线。使用Kaplan-Meier法估计EFS、OS以及相应的95%CI；通过Log-rank检验评估生存指标之间差异。当P<0.05时，认为差异具有统计学意义（双侧）。

## 2 结果

### 2.1 疗效

联合治疗组共有31例（57.4%）患者达到MPR，而单独化疗组只有5例（14.3%）患者达到（OR=8.09, 95%CI: 2.72-24.04, P<0.001）。其中联合治疗组中有25例（46.3%）患者达到pCR，单独化疗组中无pCR患者（P<0.001）。同样，两组在ypT及ypN方面也存在统计学差异（表2）。根据关键基线特征进一步分层分析，可观察到免疫治疗联合化疗更有效。但在非鳞状细胞癌中免疫治疗联合化疗的优势并不显著（OR=3.00, 95%CI: 0.53-17.16, P=0.391）。在60岁以下组、女性组及无吸烟史组中MPR也未发现统计学差异（OR=3.58, 95%CI: 0.87-14.65, P=0.100; OR=5.33, 95%CI: 0.78-36.33, P=0.175; OR=7.43, 95%CI: 1.23-45.01, P=0.050）（表3）。

**表2:** 疗效评价

Efficacy	Total (n=89)	ICIs+Chemo (n=54)	Chemo (n=35)	P
ypT, n (%)				<0.001
ypT0	27 (30.3)	25 (46.3)	2 (5.7)	
ypT1	38 (42.7)	19 (35.2)	19 (54.3)	
ypT2	14 (15.7)	6 (11.1)	8 (22.9)	
ypT3	6 (6.7)	3 (5.6)	3 (8.6)	
ypT4	4 (4.5)	1 (1.9)	3 (8.6)	
ypN, n (%)				<0.001
ypN0	53 (59.6)	41 (75.9)	12 (34.3)	
ypN1	17 (19.1)	4 (7.4)	13 (37.1)	
ypN2	18 (20.2)	8 (14.8)	10 (28.6)	
ypNX	1 (1.1)	1 (1.9)	0 (0.0)	
Pathological response, n (%)				<0.001
pCR+MPR	36 (40.5)	31 (57.4)	5 (14.3)	
PR+NR	53 (59.5)	23 (42.6)	30 (85.7)	
Radiological response, n (%)				0.001
CR	3 (3.4)	3 (5.6)	0 (0.0)	
PR	50 (56.2)	36 (66.7)	14 (40.0)	
SD	34 (38.2)	15 (27.8)	19 (54.3)	
PD	2 (2.2)	0 (0.0)	2 (5.7)	
ORR, n (%)				0.002
CR+PR	53 (59.6)	39 (72.2)	14 (40.0)	
SD+PD	36 (40.4)	15 (27.8)	21 (60.0)	

pCR: pathological complete response; MPR: major pathological response; PR: partial response; NR: negative response; CR: complete response; SD: stable disease; PD: progressive disease; ORR: objective response rate.

**表3:** MPR分层因素分析

Characteristics	Regimen	MPR, n (%)	No-MPR, n (%)	OR (95%CI)	P
Total				8.09 (2.72-24.04)	<0.001
	ICIs+Chemo	31 (86.1)	23 (43.4)		
	Chemo	5 (13.9)	30 (56.6)		
Age				7.98 (2.65-24.01)	<0.001
<60 yr	ICIs+Chemo	11 (73.3)	10 (43.5)	3.58 (0.87-14.65)	0.100
	Chemo	4 (26.7)	13 (56.5)		
≥60 yr	ICIs+Chemo	20 (95.2)	13 (43.3)	26.15 (3.10-221.02)	<0.001
	Chemo	1 (4.8)	17 (56.7)		
Gender				10.07 (3.06-33.17)	<0.001
Male	ICIs+Chemo	25 (92.6)	20 (47.6)	13.75 (2.88-65.59)	<0.001
	Chemo	2 (7.4)	22 (52.4)		
Female	ICIs+Chemo	6 (66.7)	3 (27.3)	5.33 (0.78-36.33)	0.175
	Chemo	3 (33.3)	8 (72.7)		
Smoking history				7.88 (2.63-23.62)	<0.001
Yes	ICIs+Chemo	23 (88.5)	16 (48.5)	8.15 (2.04-32.49)	0.001
	Chemo	3 (11.5)	17 (51.5)		
No	ICIs+Chemo	8 (80.0)	7 (35.0)	7.43 (1.23-45.01)	0.050
	Chemo	2 (20.0)	13 (65.0)		
Disease stage				7.75 (2.62-22.91)	<0.001
I-II	ICIs+Chemo	7 (87.5)	3 (21.4)	25.67 (2.21-298.49)	0.006
	Chemo	1 (12.5)	11 (78.6)		
III	ICIs+Chemo	24 (85.7)	20 (51.3)	5.70 (1.67-19.52)	0.003
	Chemo	4 (14.3)	19 (48.7)		
Histological type				10.28 (3.02-34.95)	<0.001
Squamous	ICIs+Chemo	25 (96.2)	19 (46.3)	28.95 (3.58-234.25)	<0.001
	Chemo	1 (3.8)	22 (53.7)		
Non squamous	ICIs+Chemo	6 (60.0)	4 (33.3)	3.00 (0.53-17.16)	0.391
	Chemo	4 (40.0)	8 (66.7)		
Cycles				8.49 (2.77-25.97)	<0.001
1-2 cycles	ICIs+Chemo	17 (81.0)	12 (36.4)	7.44 (2.03-27.28)	0.001
	Chemo	4 (19.0)	21 (63.6)		
3-5 cycles	ICIs+Chemo	14 (93.3)	11 (55.0)	11.46 (1.25-104.60)	0.022
	Chemo	1 (6.7)	9 (45.0)		

TRAEs: treatment-related adverse events. Grade 1-2 and grade 3 TRAEs were observed in three patients in the chemoimmunotherapy group. In the chemotherapy group, two patients had only grade 3 TRAEs, and the other two patients had both grade 1-2 and grade 3 TRAEs.

根据RECIST 1.1对治疗前后肿瘤影像学反应进行评价，在联合治疗组中3例（5.6%）达到完全反应缓解（complete response, CR），36例（66.7%）达到部分反应缓解（partial response, PR），15例（27.8%）达到疾病稳定（stable disease, SD）；在单独化疗组中14例（40.0%）达到PR，19例（54.3%）达到SD，2例（5.7%）达到PD。联合治疗组客观缓解率（objective response rate, ORR）显著高于单独化疗组（72.2% vs 40.0%, OR=3.90, 95%CI: 1.58-9.60, P=0.002）。

### 2.2 安全性

联合治疗组与单独化疗组中TRAEs发生率相似，分别有43例（79.6%）及30例（85.7%）患者出现任何级别的TRAEs。其中分别有3例（5.6%）及4例（11.4%）患者出现3级以上TRAEs，包括白细胞计数下降、贫血等血液系统并发症及免疫相关损伤。在单独化疗组中并未发现由于严重不良反应而终止新辅助治疗或手术延迟的病例。但在联合治疗组中1例IIIA期鳞状细胞癌患者在接受1个周期铂类+紫杉醇注射剂（白蛋白结合型）+替雷丽珠单抗的治疗后自觉全身肌肉乏力及疼痛，血液生化学检查提示肌钙蛋白I、肌红蛋白、乳酸脱氢酶、肌酸激酶及其同工酶升高。患者出现严重免疫相关心肌损伤及骨骼肌损伤，经停药、静脉输入糖皮质激素及支持治疗后好转并在用药后103 d接受根治性手术。另有1例患者经过4个周期治疗后出现轻度免疫相关肺炎及胆红素升高，静脉输入糖皮质激素治疗后好转，但手术被推迟（表4）。

**表4:** 治疗相关不良反应及分级

TRAEs	ICIs+Chemo (n=54)				Chemo (n=35)		
	Grade 1-2	Grade 3	Grade 4		Grade 1-2	Grade 3	Grade 4
Overall, n (%)	43 (79.6)	3 (5.6)	0 (0.0)		28 (80.0)	3 (8.6)	1 (2.9)
Anemia, n (%)	22 (40.7)	0 (0.0)	0 (0.0)		12 (34.3)	2 (5.7)	0 (0.0)
Increased transaminases, n (%)	12 (22.2)	0 (0.0)	0 (0.0)		11 (31.4)	0 (0.0)	0 (0.0)
Decreased neutrophil count, n (%)	10 (18.5)	2 (3.7)	0 (0.0)		6 (17.1)	1 (2.9)	0 (0.0)
Decreased appetite, n (%)	8 (14.8)	0 (0.0)	0 (0.0)		5 (14.3)	0 (0.0)	0 (0.0)
Nausea, n (%)	8 (14.8)	0 (0.0)	0 (0.0)		5 (14.3)	0 (0.0)	0 (0.0)
Increased bilirubin, n (%)	7 (13.0)	0 (0.0)	0 (0.0)		1 (2.9)	0 (0.0)	0 (0.0)
Decreased platelet count, n (%)	4 (7.4)	0 (0.0)	0 (0.0)		8 (22.9)	0 (0.0)	1 (2.9)
Rash, n (%)	3 (5.6)	1 (1.9)	0 (0.0)		2 (5.7)	0 (0.0)	0 (0.0)
Fever, n (%)	3 (5.6)	0 (0.0)	0 (0.0)		0 (0.0)	0 (0.0)	0 (0.0)
Increased blood creatinine, n (%)	2 (3.7)	0 (0.0)	0 (0.0)		0 (0.0)	0 (0.0)	0 (0.0)
Diarrhea, n (%)	1 (1.9)	0 (0.0)	0 (0.0)		0 (0.0)	0 (0.0)	0 (0.0)
Decreased adrenocortical function, n (%)	1 (1.9)	0 (0.0)	0 (0.0)		0 (0.0)	0 (0.0)	0 (0.0)
Hyperthyroidism, n (%)	1 (1.9)	0 (0.0)	0 (0.0)		0 (0.0)	0 (0.0)	0 (0.0)
Hypothyroidism, n (%)	1 (1.9)	0 (0.0)	0 (0.0)		0 (0.0)	0 (0.0)	0 (0.0)
Numbness, n (%)	1 (1.9)	0 (0.0)	0 (0.0)		0 (0.0)	0 (0.0)	0 (0.0)
Drug-related pneumonia, n (%)	1 (1.9)	0 (0.0)	0 (0.0)		0 (0.0)	0 (0.0)	0 (0.0)
Drug-related skeletal muscle injury, n (%)	0 (0.0)	1 (1.9)	0 (0.0)		0 (0.0)	0 (0.0)	0 (0.0)
Drug-related myocardial injury, n (%)	0 (0.0)	1 (1.9)	0 (0.0)		0 (0.0)	0 (0.0)	0 (0.0)

### 2.3 手术

在联合治疗组中，对于治疗前被评估为N3淋巴结转移的患者我们采用胸骨正中切口保证肿瘤的可切除性。39例（72.2%）患者接受了微创手术，4例（7.4%）患者由于肺动脉与周围组织致密粘连或需行计划外的扩大根治术转换为开胸手术；在术式方面，36例（66.7%）患者接受肺叶切除术、8例（14.8%）患者接受双肺叶切除术、4例（7.4%）患者接受袖状切除术、3例（5.6%）患者接受全肺切除术、3例（5.6%）患者接受其他术式；分别有6例（11.1%）与2例（3.7%）患者行气管及血管成形手术。单独化疗组中，12例（34.3%）患者接受了微创手术，显著低于联合治疗组（P<0.001）；共有16例（45.7%）患者行肺叶切除术、3例（8.6%）患者行双肺叶切除术、4例（11.4%）患者行袖状切除术、9例（25.7%）患者行全肺切除术、3例（8.6%）患者接受其他术式（表5）。

**表5:** 手术相关指标

Surgical information	ICIs+Chemo (n=54)	Chemo (n=35)	P
Surgical method, n (%)			<0.001
Thoracotomy	15 (27.8)	23 (65.7)	
Video-assisted thoracic surgery	39 (72.2)	12 (34.3)	
Conversion to thoracotomy, n (%)	4 (7.4)	0 (0.0)	0.151
Extent of surgery, n (%)			0.049
Lobectomy	36 (66.7)	16 (45.7)	
Bilobectomy	8 (14.8)	3 (8.6)	
Sleeve resection	4 (7.4)	4 (11.4)	
Pneumonectomy	3 (5.6)	9 (25.7)	
Others	3 (5.6)	3 (8.6)	
Bronchoplasty, n (%)	6 (11.1)	4 (11.4)	>0.999
Angioplasty, n (%)	2 (3.7)	0 (0.0)	0.517
Resection margins, n (%)			0.022
R0	51 (94.4)	27 (77.1)	
R1+R2	3 (5.6)	8 (22.9)	
Operation time, min, median (IQR)	157.5 (134.0, 213.0)	163.0 (115.0, 192.0)	0.336
Bleeding, mL, median (IQR)	100.0 (50.0, 200.0)	100.0 (50.0, 100.0)	0.758
Postoperative hospitalization, d, median (IQR)	8 (7, 11)	9 (7, 11)	0.590
Chest tube duration, d, median (IQR)	6 (4, 8)	6 (4, 7)	0.672
Postoperative complications, n (%)	24 (44.4)	16 (45.7)	0.906

VATS: video-assisted thoracic surgery.

联合治疗组R0切除率显著高于单独化疗组（P=0.022）。两组在手术时间、术中出血量、术后住院时间、引流时间及手术并发症方面均无统计学差异。常见的手术并发症为长期漏气、肺不张及胸腔积液等，未发现免疫相关手术并发症。值得注意的是单独化疗组中1例接受双肺叶切除术的患者由于血氧指数下降转入重症监护病房治疗。这可能与术前肺功能较差、手术切除范围较大及术后肺不张、胸腔积液有关。两组中均无术后30 d内死亡病例。

### 2.4 EFS与OS

联合治疗组与单独化疗组中分别有50例（92.6%）与29例（82.9%）患者接受术后个体化辅助治疗。中位随访时间为22.1个月（95%CI: 18.53-25.67），联合治疗组的中位EFS未达到，单独化疗组的中位EFS为27.2个月（复发HR=0.42，95%CI：0.19-0.92，P=0.026）。12个月时联合治疗组与单独化疗组的EFS率分别为88.2%及77.1%，24个月时分别为77.0%及56.7%。两组中位OS均未达到（死亡HR=0.32，95%CI：0.13-0.79，P=0.020）。12个月时联合治疗组与单独化疗组的OS率分别为95.9%及91.2%，24个月时分别为87.1%及67.7%（图1）。

**图1:**
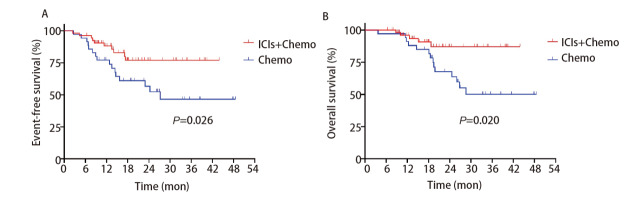
免疫治疗联合化疗及化疗组的Kaplan-Meier曲线。A：EFS；B：OS。

比较不同病理学反应，MPR组与非MPR组的EFS及OS存在显著差异。MPR组及非MPR组分别有2例（5.6%）及24例（45.3%）患者出现PD（HR=0.12, 95%CI: 0.05-0.26, P<0.001）。MPR组仅有2例（5.6%）患者死亡，1例患者死于对侧肺转移，另有1例患者死于肿瘤相关营养不良，而非MPR组有17例（32.1%）患者死亡（HR=0.16, 95%CI: 0.07-0.38, P=0.005）（图2）。

**图2:**
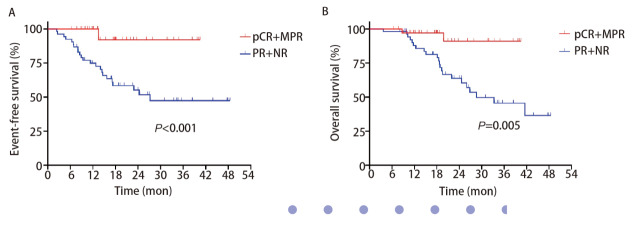
两组中关于病理学反应的Kaplan-Meier曲线。A：EFS；B：OS。

针对淋巴结病理学分期，ypN0组中位EFS未达到，ypN1组中位EFS为24.3个月（95%CI: 12.22-36.38），ypN2组中位EFS为14.0个月（95%CI: 6.35-21.65）；ypN0与ypN1组中位OS均未达到，ypN2组中位OS为26.0个月（95%CI: 16.79-35.21），三组EFS及OS显著不同（Log-rank P<0.001; Log-rank P=0.006）（图3）。

**图3:**
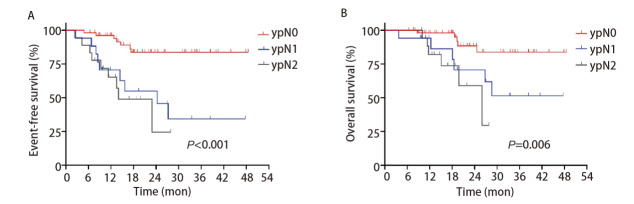
两组中关于ypN的Kaplan-Meier曲线。A：EFS；B：OS。

### 2.5 亚组分析

为进一步探究新辅助免疫治疗联合化疗用药周期对手术难度及风险的影响，根据治疗周期的不同将患者分为1-2个周期组及3-5个周期组，两组患者基线特征平衡良好。29例患者接受1-2个周期新辅助治疗，25例患者接受3-5个周期新辅助治疗。两组在R0切除率、手术时间、术中出血、术后住院时间、引流时间及手术并发症发生率方面未发现统计学差异（表6）。

**表6:** 新辅助免疫治疗联合化疗关于周期亚组分析

Items	1-2 cycles (n=29)	3-5 cycles (n=25)	P
VATS, n (%)	21 (72.4)	18 (72.0)	0.973
R0, n (%)	27 (93.1)	24 (96.0)	>0.999
Operation time, min, medain (IQR)	153.0 (132.0, 199.0)	164.0 (134.5, 228.0)	0.336
Bleeding, mL, medain (IQR)	100.0 (50.0, 200.0)	100.0 (50.0, 200.0)	0.915
Postoperative hospitalization, d, median (IQR)	7.0 (7.0, 10.5)	8.0 (7.0, 12.5)	0.380
Chest tube duration, d, median (IQR)	5.0 (4.0, 8.0)	6.0 (4.5, 9.5)	0.392
Surgical complication, n (%)	12 (41.4)	12 (48.0)	0.625

联合治疗组中位随访时间为18.7个月（95%CI: 16.70-20.70），两组的中位EFS及OS时间均未达到，且预后均无显著差异（Log-rank P=0.438; Log-rank P=0.652）。12个月时，1-2个周期组及3-5个周期组患者的EFS率分别为91.8%及83.6%，OS率分别为96.3%及95.7%；24个月时，EFS率分别为81.0%及72.5%，OS率分别为86.4%及88.8%（图4）。

**图4:**
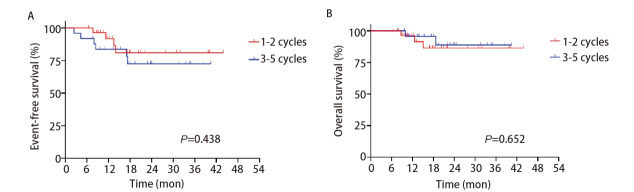
新辅助免疫治疗联合化疗关于治疗周期的Kaplan-Meier曲线。A：EFS；B：OS。

## 3 讨论

ICIs通过抑制肿瘤细胞诱导的适应性免疫抵抗^[13]^达到利用免疫机制治疗肿瘤的目的，在治疗包括恶性胸膜间皮瘤在内的多种晚期恶性肿瘤方面取得显著效果^[14⇓-16]^；另一方面术前应用ICIs也可获益^[6,17]^。本研究通过回顾性收集NSCLC真实世界数据，对比术前应用免疫治疗联合化疗与单独化疗的有效性与安全性，并通过随访患者比较两组EFS及OS。

MPR可作为评估NSCLC新辅助治疗的短期研究终点^[18]^。III期临床研究CheckMate-816^[6]^报道MPR率及pCR率分别为36.9%及24.0%。2023年，美国癌症研究学会（American Association for Cancer Research, AACR）及美国临床肿瘤学会（American Society of Clinical Oncology, ASCO）会议上分别首次公布了III期临床研究AEGEAN^[19]^及Neotorch的数据，二者均采用术前新辅助免疫治疗联合化疗，术后辅助单独免疫治疗的模式，其MPR率分别为33.3%及48.5%，pCR率分别为17.2%及24.8%^[20,21]^。本研究显示，在EGFR/ALK野生型患者中，免疫联合治疗MPR及pCR的比例分别为57.4%及46.3%，单独化疗分别为14.3%及0.0%，高于III期临床实验，这可能与我们在数据收集阶段只回顾性收集手术患者有关。可见，相比于单独化疗，术前联合应用免疫治疗能显著提高患者的病理缓解率。通过对MPR患者及非MPR患者基本特征的进一步分析，我们发现新辅助免疫治疗联合化疗对鳞状细胞癌的效果优于非鳞状细胞癌，但未发现统计学差异（OR=3.00, 95%CI: 0.53-17.16, P=0.391），这与CheckMate-816相关结论一致。而未在年龄<60岁、无吸烟史及女性三组中发现二者的统计学差异，这可能与肺腺癌较鳞状细胞癌更易发生于此三类患者有关^[22,23]^。

两组未在TRAEs及严重不良反应发生率方面发现统计学差异。研究期间我们观察到1例患者出现3级免疫相关心肌及骨骼肌损伤，1例患者出现轻度免疫相关肺炎，二者经糖皮质激素治疗及停药后均好转。因此，新辅助免疫治疗联合化疗的安全性仍值得肯定，但治疗时仍需动态监测肌钙蛋白、肌红蛋白、肌酸激酶及其同工酶等指标变化。

相比于单独化疗，联合免疫治疗并未明显增加手术难度与术后并发症发生率。同时，免疫治疗联合化疗显著提高了R0切除率；在术式方面接受肺叶切除术的患者比例更高，缩小了手术切除范围，为患者保留了肺功能，有利于患者术后恢复及生活质量的提高。

Impower130研究^[24]^显示，相比于化疗，EGFR/ALK基因突变阳性患者的OS无法从Atezolizumab联合化疗中获益（HR=0.98, 95%CI: 0.41-2.31）。我们发现在EGFR/ALK野生型患者中，新辅助免疫治疗联合化疗的EFS及OS显著优于单独新辅助化疗。本研究的中位随访时间为22.1个月（95%CI: 18.53-25.67），12及24个月的EFS分别为88.2%及77.0%，较CheckMate-816的相关数据高，这种差异可能由术后辅助治疗方案不同造成。相比于CheckMate-816的辅助化疗或者放射治疗，本研究针对不同患者术后采用个体化治疗方式，包括免疫治疗联合化疗、单独免疫治疗、放射治疗等。IMpower010研究^[25]^对PD-L1≥1%患者术后辅助单独免疫治疗进行了肯定。NADIM首次将新辅助免疫治疗联合化疗与术后辅助免疫治疗结合，42个月时PFS率及OS率分别高达69.6%与78.9%^[26,27]^。国际会议上公布的III期临床试验AEGEAN及Neotorch^[20,21]^也同时将术后辅助免疫治疗纳入研究，使NSCLC患者在术后长期获益。

对于新辅助化疗，肿瘤病理反应及ypN分期决定了患者的长期预后^[18,28]^。因此，我们根据肿瘤病理学反应及ypN分期的不同进行预后分析，发现MPR组较非MPR组、ypN0较ypN+患者的EFS及OS显著延长。这也证实对于新辅助免疫治疗联合化疗，MPR及ypN0仍是影响患者预后的主要因素。

neoSCORE研究^[29]^显示新辅助Sintilimab联合化疗3个周期较2个周期MPR及pCR率分别提高14.5%（P=0.260）及4.9%（P=0.660），且未增加手术难度及风险。同样，我们在亚组分析中发现治疗周期的增加并未显著提高手术难度与术后并发症发生率。二者的EFS及OS未发现统计学差异，但可能受到术后个体化辅助治疗的影响，相关数据仍需进一步随访跟进。

本研究也存在一定的局限性。第一，为回顾性研究且样本量较小。第二，随访时间较短。第三，免疫治疗联合化疗方案不一致。

综上，在手术患者中，相比于术前新辅助化疗，新辅助免疫治疗联合化疗显著提高MPR率及pCR率，且未增加手术难度及治疗相关不良事件发生率。2年EFS及OS支持术前新辅助免疫治疗联合化疗的有效性。新辅助免疫治疗联合化疗周期的增加对手术难度及术后并发症发生率并无显著影响。

**Acknowledgments**

The authors thank Yongxin ZHANG of Anshan Cancer Hospital for collecting the data and the Liaoning Province People's Livelihood Science and Technology Program Joint Program for funding. We thank International Science Editing (http://www.internationalscienceediting.com) for editing the English text of this manuscript.

**Competing interests**

The authors declare that they have no competing interests.
